# News and Events of the Week

**Published:** 1910-11-05

**Authors:** 


### News and Events of the Week.

Mr. Haldane, in addressing the Scottish branch of the British Red Cross Society last week, emphasised the importance of the medical service to the Army. "The wastage," he remarked, "is very great if there is no proper medical service."

Dr. James Kingston Fowler (Senior Physician to the Middlesex Hospital) and Mr. Alfred Pearce Gould (Senior Surgeon to the Middlesex Hospital) were received by the King last week, when his Majesty conferred upon them the honour of Knighthood and invested them with the Insignia of a Knight Commander of the Royal Victorian Order.

Twenty doctors and clergymen, the former being not all members of the Church of England, met in the Chapter House of St. Paul's Cathedral last week to discuss the relation between the practice of medicine and the Church's ministry to the sick. Archdeacon Sinclair presided, and Mr. Geoffrey Rhodes, editor of the book of essays entitled "Medicine and the Church," of which we hope shortly to publish a review, acted as secretary. One of the objects of the promoters was to emphasise the importance of spiritual help in illness; but another was to warn the public against persons attempting to heal under the cloak of religion.

Lectures at the Royal College of Physicians.—The Bradshaw Lecture on "The Results of Bronchial Obstruction" will be delivered by Dr. G. Newton Pitt on November 1. Sir T. Clifford Allbutt, Regius Professor of Physic at Cambridge, will give the FitzPatrick Lectures on November 3 and 8, the subject being "Greek Medicine in Rome." The Horace Dobell Lecture on "The Problem of Pulmonary Tuberculosis considered from the Standpoint of Infection" will be delivered by Dr. W. Bulloch on November 10. The following lectures will be delivered in 1911: The Milroy, by Dr. A. E. Boycott; the Goulstonian, by Dr. Hertz the Lumleian, by Dr. J. M. Bruce; the Oliver-Sharpey, by Dr. J. Mackenzie; the Croonian, by Dr. H. Head; and the FitzPatrick, by Dr. Raymund Crawford.

The Right Hon. Sir Savile Crossley, Bart., P.C., K.C.V.O., will be the guest of the evening at the annual dinner of the Incorporated Association of Hospital Officers, to be held at the Gaiety Restaurant, on Saturday, November 26, at 7 p.m.

The Radium Institute created by the Academy of Sciences at Vienna, says the *Times* correspondent, has been formally opened by the Archduke Rainer. The new institute is to be devoted solely to chemical and physical research, and will be open to scientific men of all countries. The institute has at its disposal three grammes of radium from Joachimstal.

Lord Northcote has addressed an appeal to the Lord Mayor in favour of the allocation of a part of the fund raised for a London memorial to the late King to establish an Edward VII. Tropical Research Fund. The scheme he supports, for the protection of human life in the tropics, is not desired by its distinguished promoters "to take precedence of all other schemes, but it is hoped that some portion, at any rate, of the total fund will be devoted to it."

Dr. Colman has decided to give up his work at St. Thomas's Hospital. When he joined the staff twelve years ago he resigned his appointment of physician to the National Hospital, Queen Square, while retaining that of physician to the Hospital for Sick Children, Great Ormond Street, but the pressure of work involved by two hospital appointments and by private practice has been very great, and after endeavouring to find some other way of lessening his work he has reluctantly felt compelled to take this step. "Dr. Colman," remarks the *Hospital Gazette*, "will be greatly missed by his colleagues for his faithful performance of his share of the hospital and school work, and for his unstinting help in committee work behind the scenes, which means so much in the smooth management of a hospital and medical school; but we understand that a way is being found through which his sound judgment of men and matters will still be at our service."

A Conference on the Feeding of Nurses will be held at Caxton Hall to-day (Saturday), at 2,30 p.m. Matrons desiring to be enrolled as members of the Conference, and individual nurses and others interested, wishful to obtain visitors' tickets, should make early application to the Secretary, National Food Reform Association, 178 St. Stephen's House, Westminster, since the accommodation is limited.

Mr. John Langton, F.R.C.S., of Bentinck Street, Cavendish Square, London, W., who died on September 11, left estate of the gross value of £8,777 12s. 11d., of which the net personalty has been sworn at £6,731 10s. 7d.

The Tuberculosis Exhibition will be opened in the Exhibition Buildings, York, on November 7, by Lady Nunburnholme at 3.30 p.m. The Exhibition, which comprises exhibits lent by the National Association for the Prevention of Consumption and by the Women's National Health Association of Ireland, and special sections devoted to home and school hygiene and to local exhibits, will be open, free of charge, for a week. Popular lectures and short talks will be the order of the evening while the Exhibition is open.

At the last meeting of the London County Council, the Public Control Committee recommended that Mr. S. I. Oddie, Deputy Coroner for West London, be appointed Coroner for the Central District, at a salary of £1,250 a year, in succession to the late Dr. Danford Thomas. Mr. T. C. E. Goff, Chairman of the Committee, said the Committee was fully cognisant of the good work done by Mr. Walter Schröder, who had acted as deputy to Dr. Danford Thomas for many years. Unfortunately, if the Committee had to carry out the Departmental Committee's recommendation, Mr. Schröder had no qualification, either medical or legal, and that weighed very largely in making the selection. Mr. W. Haydon, Vice-Chairman of the Committee, moved, as an amendment, that Mr. Schröder be appointed. For thirty-nine years, he said, Mr. Schröder had been connected with Coroners' Courts, and had conducted practically all the inquests in the central district in recent years. He had held over 9,000 and attended 20,000 inquests, and had an unrivalled asset in his vast experience. The Vice-Chairman added that it was understood that if Mr. Schröder were appointed, and were called on to resign in ten years' time at the age of sixty-five, he would not claim a pension. The amendment, after some discussion, was carried by 52 votes to 17. A motion to impose a condition that Mr. Schröder should not claim a pension on retirement was defeated by 34 votes to 27. Mr. Schröder was forthwith introduced, and took the oath of office.

